# Report on 565 Cases of Eye Diseases Treated at the Central Free Dispensary

**Published:** 1878-01

**Authors:** William T. Montgomery

**Affiliations:** Chicago


### REPORT ON 565 CASES OF EYE DISEASES TREATED AT THE CENTRAL FREE DISPENSARY.

By William T. Montgomery, M.D., Chicago.

*(Read before the Chicago Medical Society Dec. 3, 1877.)*

During the past two years I have treated at the Central Free Dispensary, 565 patients affected with diseases of the eye. We had 108 affections of the lids; 24 of the lachrymal apparatus; 192 of the conjunctiva; 138 of the cornea; 31 of the iris and choroid; 26 of the lens; 18 of refraction and accommodation; 12 of optic nerve; 12 of the muscles; and 4 of the globe.

Of the 108 affections of the lids there were: eczema, 14; blepharitis ciliaris, 59; hordeolum, 6; ulcer of lids, 4; trichiasis, 13; entropium, 4; chalazion, 8.

I shall only notice one disease of this group as being of interest to the general practitioner. Blepharitis ciliaris comprises more than half of the whole number of lid affections, 59 in 108. This is essentially an ulcerative inflammation of the edges of the lids and ciliary follicles. As impure air, uncleanliness, and improper nourishment, are common causes of blepharitis, we have in the bad hygienic conditions of most dispensary patients, elements favorable for its production. Of the 59 cases here presented, 37 were under 10 years of age, and a large per cent. of the whole number presented evidences of the strumous diathesis. Tonics entered largely into the treatment of these cases. Quinine, iron, and cod liver oil were the favorite remedies. The local treatment was as follows: thorough cleansing of the edges of the lids, and keeping them free from the crusts which form so rapidly in this affection. When these were thick and firm, I either removed them in the first instance myself, or ordered warm fomentations until they were softened, and were easily removed by the attendants. Some form of mercurial ointment (usually the red oxide) was prescribed, to be applied to the edges of the lids night and morning. In some of the severer and more obstinate cases, the edges of the lids were brushed with the nitrate of silver solution, grs. xx. to xl. to ℥ i., two or three times a week. Of the 24 affections of the lachrymal apparatus, there were eversion of puncta, 12; abscess of lachrymal sac, 3; stricture of nasal duct, 9. I will not occupy your time with either of these.

The 192 conjunctival affections were as follows: catarrhal conjunctivitis, 122; granular conjunctivitis, 45; phlyctenular conjunctivitis, 8; purulent conjunctivitis, 6; infantile conjunctivitis, 1; pterygium, 7; burn of conjunctiva, 2; epithelioma of conjunctiva, 1.

Catarrhal conjunctivitis numbers more than half of the diseases of the conjunctiva here presented, 122 in 192. Of the whole list of eye diseases, none yield more readily to proper treatment than does this, but neglected or improperly treated, it may become chronic and obstinate. In the milder cases, I have generally brushed the conjunctiva with a gr. iii. to ℥ i. sol. of silver nitrate, and prescribed gr. ii. sol. of sulphate zinc, to be dropped into the eyes two or three times daily. This, together with cleanliness, and protecting the eyes from all causes of irritation, was all the treatment required in these milder cases.

For the more severe cases, a stronger solution of nitrate was used; but seldom stronger than gr. v. to the ℥ i. This was applied once daily, and the patient given a weak astringent solution to use at home once or twice daily. To prevent the gluing together of the lids, the patient was directed to grease the edges at night with fresh lard or cream. If the edges of the lids were excoriated, the red oxide ointment was ordered.

I do not wish to convey the impression that nitrate of silver is the only remedy I have used in the treatment of catarrhal conjunctivitis, but I must say it is the best remedy I have used in this disease, as well as in purulent conjunctivitis.

Acetate of lead, gr. v. or gr. x. to ℥ i., is the next most efficient remedy in catarrhal conjunctivitis.

The patient's general and hygienic conditions were made as good as possible.

Of the 192 conjunctival affections, there were 45 cases of granular conjunctivitis. This is essentially a disease of adult life. Of the 45 cases, but one was under 15 years of age, 5 over

50. Some eminent authorities have maintained that it is frequently due to constitutional causes. This has not appeared to be true of the cases here presented. While I am not able to give the exact number of cases which presented any evidence of the scrofulous or other diathesis, I am sure it was not sufficient to enter into account as a cause. It is true a large per cent. of these cases were in a weakened debilitated condition, but I am inclined to adopt the views of Soelberg Wells; "that the ill-health is often, rather the effect than the cause, for the very protracted course of the disease is sure to tell more or less severely upon the health and spirits of the patient."

As to the treatment of granular conjunctivitis, I haven't anything very encouraging to offer. This disease has long been, and still remains, an eye-sore to the profession. But since the pathology of the disease has become better understood, the treatment, if not more successful, has been less harmful. I think it is unanimously conceded now, that true granulations are not simply enlarged papillæ, but new formations, neoplastic growths. And that the object of treatment is not to burn out, or chemically destroy them, but to keep up sufficient irritation or inflammation to cause their absorption.

The remedies which have been recommended and used with more or less benefit in the treatment of this disease, include almost the entire list of caustics, irritants, and astringents. I do not consider the choice of remedies as important as the proper application of the remedies chosen. Yet, it is perhaps true, that every one who has had any considerable experience in the treatment of granular conjunctivitis, has settled upon some favorite remedy. I must admit that this is true with myself, and the local application I have used most in the treatment of these cases, is what I shall term the sulphate solution. I first began to use this solution over two years ago, at the suggestion of my friend Dr. Holmes. The following is the formula: Ferri sulph., zinci sulph., cupri sulph., and alum. sulph., equal parts in distilled water. A ʒ i. of each to f℥ i., makes a saturated solution. I keep a solution of this kind, and dilute it as the case may require, occasionally using it full strength. While the strong solution is a pretty severe application, and requires to be used cautiously, it is not caustic, and the pain it produces, though more severe for a few minutes, does not last as long as that produced by the application of blue stone.

I have generally used a solution containing gr. v. of each to ℥ i. of distilled water, applying with a brush once daily.

A large per cent. of the cases of chronic granulations have more or less vascularity or roughness of the cornea, and it is in these cases that I have found this solution especially useful. I seldom use the saturated solution, but in cases with dense pannus, great intolerance of light and spasm of the lids have found happy effects from its use. I do not present this solution as a specific for granulations, or as being the only remedy I use in the treatment of this disease, but as being the best application I have used. The next best remedies are blue stone, red or yellow oxide of mercury ointment, nitrate of silver, and acetate of lead in the order named. Quiniæ sulph. dusted on the conjuntiva once daily, I have found to act happily in relieving photophobia and spasm of the lids, but have not observed that it has any effect upon the granulations.

I have looked upon the affection as purely local, but have tried to improve the general condition where I found it bad.

We have 134 corneal affection, as follows: ulcer of cornea, 19; wound of cornea, 11; abcess of cornea, 6; mote in cornea, 3; phlyctenular keratitis, 47; vascular keratitis, 12; interstitial keratitis, 6; leucoma, 13; staphyloma, 5; pannus, 12.

Of this group we shall only notice phlyctenular keratitis, which comprised nearly one-third of the whole number of corneal troubles. This number I think fairly represents the relative frequency with which the disease is met, and its relative importance to the physician.

Phlyctenular inflammation of the cornea, so far as the cases here presented are concerned, is as much a children's disease as any that is classed as such. The books on diseases of the eye tell us that "this is a disease most frequently met with in children from six to twelve years of age." Of the number here presented, thirty-two were under six years of age, and only three were over twelve. This disease has been termed by some, and not inappropriately, scrofulous corneitis.

If there is one eye disease met with in dispensary practice, that more imperatively than any other demands attention to the general condition it is this. Indeed, so strongly have I been impressed with this truth that I have almost fallen into the routine practice of prescribing tonics in every case of this affection.

My favorite remedies are the syrup of the iodide of iron and cod-liver oil, together with as nourishing food and good hygienic conditions as possible. If the little patient is unable to take the pure oil, I have lately been in the habit of prescribing Scott's emulsion, which is easily taken, and is an excellent substitute for the pure oil.

Quinine and Fowler's solution of arsenic are useful tonics in this disease. The latter is more especially indicated in cases accompanied with an eczematous rash of the face. If it is important to attend to the general health in this disease, we must not do so to the neglect of the local treatment. We sometimes get wonderful results from local treatment alone. But to successfully combat that most harrassing feature of the trouble—the great tendency to recurrent attacks of the inflammation—we must be on the alert with both general and local treatment. I need not remind those who have had any experience with phlyctenular keratitis, that the most striking characteristics of the disease are the photophobia, blepharospasmus, and general nervous irritability of the patient. Indeed these symptoms are almost pathognomonic, and, when present in a marked degree, the local remedy is atropine. My practice has been to prescribe atropiæ sulph. gr. ii. to ℥ i. of distilled water, to be dropped into the eye once in two hours, until the pupil is fully dilated, and three or four times a day afterward. If the spasm of the lids is very great, I make the first applications myself, and charge the parents to make sure of getting the drops in the eye every time at home. The effect of atropine in this disease is sometimes truly marvelous. It acts as a local anæsthetic upon the cornea, relieving the irritability of this structure and the ciliary nerves. It also diminishes the intraocular tension, and, by thus lessening the pressure upon the cornea, favors its nutrition and reparation.

As soon as the irritability is relieved, we begin to dust upon the cornea once daily, the mild chloride of mercury. This, besides hastening the absorption of opacities remaining, is one of the most efficient remedies we have in preventing relapses of the inflammation. The red or yellow oxide of mercury gr. ii. to ʒ i. of lard or glycerole of starch are equally good, but are not so convenient for me to use.

Of the 31 affections of the iris and choroid, there were 19 cases of iritis; kerato-iritis, 4; irido-choroiditis, 7; and glaucoma, 1. We can only very briefly refer to one of this group—iritis. While this disease is not so frequently met with as the others referred to, it is important, from the fact that neglect or improper treatment is more apt to produce permanent injury to the eye.

Iritis is not a favorable disease to treat in dispensary practice, because two important factors in its treatment—rest of the eyes and exclusion of light—are denied.

It is not long since mercury was considered almost a *sine qua non* in the treatment of this disease. If there is anything in medicine I am conservative in, it is in the internal use of this drug. I make it a point never to administer it in the treatment of iritis, unless there is evidence of specific trouble, until I have given local medication a thorough trial.

In the treatment of these cases, I do not remember a single case of non-specific iritis that did not yield without the use of mercury.

As a local application, we have in atropine a remedy that in most cases of iritis meets all the indications. 1st. It dilates the pupil, and prevents adhesions between the iris and capsule of the lens. 2d. It paralyzes the accommodation, and puts the eye in a state of rest. 3d. It lessens the eye tension, and thus relieves the intra-ocular circulation and diminishes the congestion of the iris. 4th. It relieves pain.

But to get the full effect, a strong solution must be used, at least gr. iv. of the sulphate of atropia to the ℥ i. of distilled water. I try to see the patient once a day, and drop the solution into the eye myself, four or five times, at intervals of a few minutes, and direct the patient to use it at home every hour. After the eye is well under the influence of the atropine, four or five times a day is often enough to use it. If the pain is severe, hot fomentations applied to the eye is my first additional resort; second, leeches to the temple; and third, morphia. The eyes are protected from the light, and other sources of irritation, as well as possible. The patient's bowels are kept regular; the skin and kidneys active.

Of the remaining eye affections, we have cataract, 24; dislocation of lens, 2; neuro-retinitis, 4; atrophy of optic nerve, 4; amblyopia, 4; asthenopia, 4; myopia, 4; hypermetropia, 6; strabismus convergent, 7; divergent, 1; paralysis of third nerve, 1; paralysis of sixth nerve, 3; panophthalmitis, 2; atrophy of globe, 1; tumor of orbit, 1.

